# Design and Performance Evaluation of a Novel Wearable Parallel Mechanism for Ankle Rehabilitation

**DOI:** 10.3389/fnbot.2020.00009

**Published:** 2020-02-18

**Authors:** Shiping Zuo, Jianfeng Li, Mingjie Dong, Xiaodong Zhou, Wenpei Fan, Yuan Kong

**Affiliations:** ^1^College of Mechanical Engineering and Applied Electronics Technology, Beijing University of Technology, Beijing, China; ^2^Beijing Institute of Control Engineering, Beijing, China

**Keywords:** ankle rehabilitation, parallel robot, mechanical design, performance indices, performance evaluation

## Abstract

Repetitive and intensive physiotherapy is indispensable to patients with ankle disabilities. Increasingly robot-assisted technology has been employed in the treatment to reduce the burden of the therapists and the related costs of the patients. This paper proposes a configuration of a wearable parallel mechanism to supplement the equipment selection for ankle rehabilitation. The kinematic analysis, i.e., the inverse position solution and Jacobian matrices, is elaborated. Several performance indices, including the reachable workspace index, motion isotropy index, force transfer index, and maximum torque index, are developed based on the derived kinematic solution. Moreover, according to the proposed kinematic configuration and wearable design concept, the mechanical structure that contains a basic machine-drive system and a multi-model position/force data collection system is designed in detail. Finally, the results of the performance evaluation indicate that the wearable parallel robot possesses sufficient motion isotropy, high force transfer performance, and large maximum torque performance within a large workspace that can cover all possible range of motion of human ankle complex, and is suitable for ankle rehabilitation.

## Introduction

As the population ages, increasingly more individuals experience ankle disabilities caused by stroke and cerebral palsy, which may lead to lack of mobilization, irregular pain of body, insufficient capacity to support weight, and chronic joint instability. During the conventional manually physiotherapy, human ankle complex (HAC) is moved by a physical therapist with its range of motion (ROM). However, it possesses many limitations such as, the duration inconsistency and frequency indetermination of the treatment procedures, the physical demand, and experience requirement of the therapist, and the subjective evaluation of the therapeutic results (Meng et al., 2015; Hussain et al., 2017). In view of this situation, to provide high-quality rehabilitation treatment with repetitive sessions, quantitative measurements, scientifical therapy, and systematic operation, robot-assisted rehabilitation has become a field that receives more, and more research attention. To date, various ankle rehabilitation devices have been introduced based on different concepts that can be mainly divided into two categories: ankle exoskeleton and parallel platform-based robots.

Focusing on walking gait treatment on treadmill or over-ground, ankle exoskeletons are wearable with mechanical parts attaching to the human limb. A typical instance is the active ankle-foot orthosis proposed by Blaya and Herr (2004), by employing series elastic actuator (SEA), rotary potentiometer, and ground reaction force sensors, a gait pathology known as drop-foot can be treated via variable-impedance control. Similarly, exoskeletons (Kim et al., 2011; Zhu et al., 2011; Lopez et al., 2013; Meijneke et al., 2014; Witte et al., 2015; Dijk et al., 2017; Erdogan et al., 2017) were also developed with the concept of SEA to provide push-off assistance. Moreover, robotic tendons, i.e., a DC motor in series with a spring, were used in ankle-foot orthoses (Hollander et al., 2006; Boehler et al., 2008; Oymagil et al., 2008; Ward et al., 2011) to provide sufficient energy and peak power saving for systems; pneumatic muscle actuators (PMA), due to their intrinsically compliant and high power/weight ratio, were also widely selected as the actuation technology of the ankle exoskeletons (Ferris et al., 2006; Gordon et al., 2006; Kinnaird and Ferris, 2009; Sawicki and Ferris, 2009; Park et al., 2014). Additionally, directly aligning several types of actuators, including rotating actuator assembly (Ren et al., 2017), servo motor (Yoshizawa, 2010; Yao et al., 2018) and bidirectional pneumatic actuator (Shorter et al., 2011), to the joint axis is another option for researchers.

The aforementioned exoskeletons possess one degree of freedom (1-DOF) for assisting plantarflexion/dorsiflexion (PL/DO) motion. By applying parallel mechanism-based design, more DOFs can be performed by exoskeletons. A well-known example is the Anklebot proposed by Roy et al. (2009). Two linear actuators were arranged in parallel to aid recovery of PL/DO and inversion/eversion (IN/EV), while the adduction/ abduction (AD/AB) can be achieved via the rotation of the leg. Subsequently, a scaled down version called pediAnklebot (Michmizos et al., 2015) is developed for pediatric rehabilitation. Fan and Yin (2009) presented an ankle exoskeleton with a 3-RPS (revolute-prismatic-spherical) parallel mechanism as the main mechanical structure in cooperation with an electromyographic-based neuro-fuzzy controller. A reconfigurable ankle exoskeleton (Erdogan et al., 2017) was proposed for multiple phases of treatment, in which the 3-RPS structure and the 3-UPS (universal-prismatic-spherical) structure can be interconverted via lockable joints. Stewart platform (Takemura et al., 2012; Nomura et al., 2015) was also utilized for ankle exoskeletons.

In the early stage of rehabilitation, the movements of ankle are weak and stiff due to the muscle atrophy or the loss of physiological muscles synergies. Thus, before performing gait treatment using wearable exoskeleton, a parallel platform-based robot, due to the features of superior adaptability, excellent accuracy and high stiffness, is a more suitable option to ensure reliability and safety in the process of rehabilitation treatment. Moreover, by fixing the foot on the platform, three rotational DOFs (i.e., PL/DO, IN/EV, and AD/AB) of the HAC can be all provided treatment.

Girone et al. (1999) proposed a pneumatically actuated ankle rehabilitation robot (Rutgers Ankle) with a force feedback system. A virtual reality environment has been developed to make rehabilitation more effective and enjoyable. By employing passive central struts in the mechanical structure to determine the number of DOFs and increase the payload capability, lower-mobility parallel robots (Dai and Zhao, 2004; Liu et al., 2006; Saglia et al., 2009) have been proposed for ankle rehabilitation with their DOFs better matching with that of the HAC.

Tsoi et al. (2009) replaced the central strut with the lower-limb of the patient (i.e., the HAC is directly adopted to constrain the motion of the platform). In comparison of the aforementioned platform-based robot, this robot realizes an aligned rotation center between the platform and the HAC in the process of rehabilitation. To avoid the safety issue caused by excessive load in this method, physical rotation axes (i.e., kinematic constraint mechanisms) were specially designed. Specifically, Jamwal et al. (2014) proposed a compliant parallel robot by arranging four PMAs parallel to the shank of the patient. Three bearings were setting into the platform as kinematic constraint. Thanks to the inherent muscle-like behavior, compliant motions can be achieved during different treatment modes with the help of a fuzzy logic controller. Analogous to Jamwal et al. (2014), in Zhang et al. (2017), The University of Auckland developed the other compliant robot powered by four PMAs that arranged in a tilted manner. A three-linkage serial mechanism was set as the kinematic constraint of this robot, and the connection points (i.e., spherical joints) can be adjusted along certain directions to achieve reconfigurable workspace and torque capacity. By selecting two types of identical active branches, i.e., 3-UPS structure and 3-RUS (revolute-universal-spherical), to produce obliquity of the platform and using serial equivalent spherical mechanisms to satisfy all 3-DOF rotational ankle rehabilitation, actuated parallel mechanisms introduced by Wang et al. (2013, 2015) are another typical instance.

Notably, the arrangement of the physical rotation axes has become an effective method for a parallel platform-based ankle rehabilitation robot to actualize the required treatment action, realize aligned rotational center and ensure primary safety. Redundant actuation technology, despite having received widespread application, may present a complicated structure and control scheme, and then increase the manufacturing and operation cost. Moreover, a totally relaxed lower-limb may prevent the HAC from fully stretching into the extreme position, which limits the improvement and functional recovery of muscle strength. Meanwhile, repeatedly changes in sitting posture caused by a loose shank may lead to re-injury to the patient. This paper put forth a novel parallel robot for ankle rehabilitation with a wearable design concept to provide maximum safety protection. A simple configuration is adopted to realize actuator non-redundancy and reduce the relatively cost.

The remainder of this paper is organized as follows: The HAC anatomy and configuration design are presented in section HAC Anatomy and Configuration Design. In section Kinematic Analysis, the kinematic analysis, including inverse position solution and velocity Jacobian matrices, are derived, based on which several performance indices are defined in section Performance Indices. Section Mechanical Design describes the mechanical design in detail. Section Performance Evaluation analyzes the performance including reachable workspace, motion isotropy, force transfer performance and maximum torque performance. Finally, we discuss the main findings and draw the main conclusions of the study.

## Materials and Methods

### HAC Anatomy and Configuration Design

**Figure 1:**
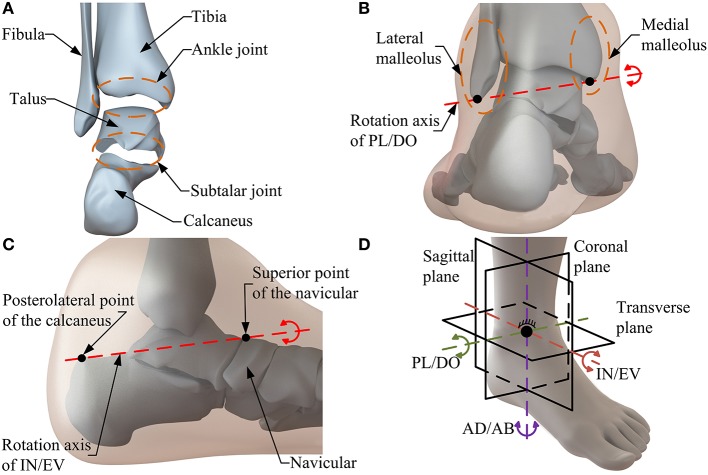
The HAC anatomy. **(A)** The structure of the HAC. **(B)** Rotation axis of PL/DO. **(C)** Rotation axis of IN/EV. **(D)** Motion form of the HAC.

Considering HAC anatomy in the design process of an ankle rehabilitation robot is a basic guarantee to ensure the comfort and safety of patients during rehabilitation. Thus, it is necessary to carry out the anatomical analysis before determining the configuration of the robot. As one of the most complicated joint in the human body, the HAC (Figure 1A) contains two anatomically separate joints, namely, the ankle joint and the subtalar joint (Dai and Zhao, 2004; Khalid et al., 2015). Specifically, the ankle joint consisting of the tibia, fibula and talus, is located above the subtalar joint which is formed by the talus inferiorly and the calcaneus superiorly (Dai and Zhao, 2004). Moreover, three rotational motions, i.e., PL/DO, IN/EV, and AD/AB, resulted from the interaction between the articulating joint surfaces and the constrained ligament constitute the basic motion form of the HAC (Isman and Inman, 1969). The rotation axis of the ankle joint (i.e., PL/DO) passes through the tips of the medial and lateral malleolus (Figure 1B), and the orientation of IN/EV (i.e., the rotation axis of the subtalar joint) is approximated by the line between the superior point of the navicular and the posterolateral point of the calcaneus (Figure 1C; Dul and Johnson, 1985; Dettwyler et al., 2004). The combined motion of the ankle joint and the subtalar joint, as well as the rotation between the tibia and fibula contribute to the AD/AB (Khalid et al., 2015). In biology, the aforementioned skewed rotation axes produce rotational motion in all three orthogonal planes (i.e., sagittal, coronal, and transverse planes; Feuerbach et al., 1994). Thus, when considering the kinematic model of the HAC from the perspective of mechanism, the two separate subjoints can be simply regarded as a 3-DOF spherical joint in a combined manner (Figure 1D).

**Figure 2:**
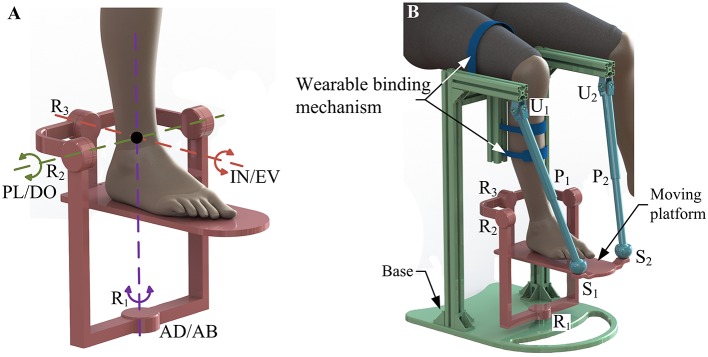
**(A)** Constraint branch. **(B)** Human-robot system.

According to the kinematic model of the HAC, a serial constraint branch (Figure 2A) with a three axes-intersected revolute joints (R_1_, R_2_, and R_3_) structure is selected as an equivalent spherical joint to imitate the 3-DOF rotational motion of the HAC and determine the rotation center of the robot. By using this constraint branch, the ankle rehabilitation treatment can be performed under a human-robot compatible situation with fixed rotation center and precise DOFs. Moreover, two identical non-constraint rods with UPS structure are selected as the kinematic branches. Based on the aforementioned consideration, a 2-UPS/RRR parallel mechanism is proposed as the basic configuration of the ankle rehabilitation robot. Joints P_1_, P_2_, and R_1_ are active, whereas all others are passive. For patients with ankle disabilities, the movement of the HAC becomes weaker and stiffer, and even with ankle spasticity/contracture (Zhou et al., 2014). Thus, to protect the already fragile ankle from secondary injury, the position of the shank and the HAC should remain stationary with respect to the foot. Additionally, a follow-up shank makes it difficult to perform treatment at the extreme position of the HAC, thus affecting the full recovery of function. By inverting the 2-UPS/RRR parallel mechanism (i.e., the base is positioned above the moving platform), and inserting the shank of the patient into the mechanism as a part of the base via an accessory wearable binding mechanism, decoupled foot-shank motion (i.e., the shank will not move with the foot) and the maximum safety guarantee can be achieved during different treatment modes.

The formed human-robot system consisting of the HAC and the wearable parallel robot is presented in Figure 2B. Analogous to the known Tricept mechanism, two unconstrained UPS branches provide six DOFs to the moving platform while three constraint force line vectors that through one point in space are acted in the platform wrench system via the properly constrained RRR branch, and thus retain three rotational ones of six DOFs. The line graph of the constraint spaces and freedom spaces of the branches and moving platform is presented in Table 1 based on Grassmann line geometry, in which the green solid lines, green solid double arrow lines, and blue dotted lines indicate the rotational DOF, translational DOF, and constraint force, respectively.

**Table 1:** Line graph of the constraint spaces and freedom spaces.

**Structure**	**Constraint space**	**Freedom space**
UPS branches	No constraint	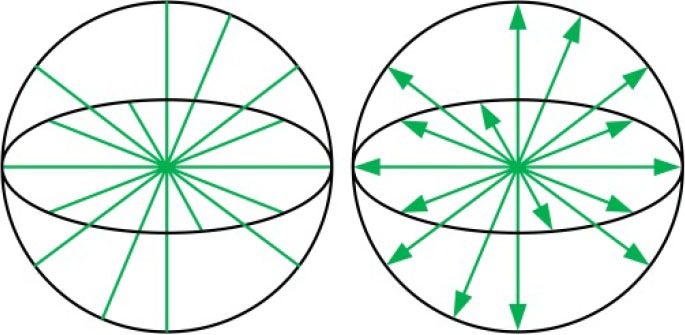
		Three-dimensional rotation and three-dimensional translation
RRR branch	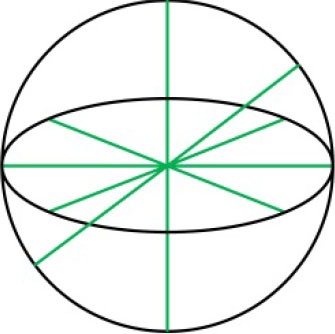	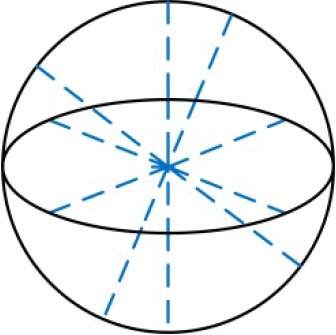
	Three-dimensional constraint force	Three-dimensional rotation
Moving platform	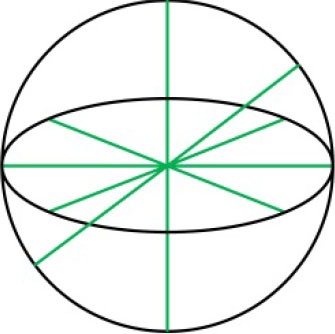	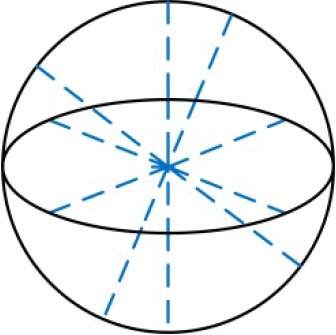
	Three-dimensional constraint force	Three-dimensional rotation

The K-G formula (Huang et al., 2013) is used to verify the number of DOFs obtained from the aforementioned analysis. The wearable parallel robot consists of a base, a moving platform, a constraint branch and two kinematic branches:

(1) $$\begin{array}{l} F=6\left(n-g-1\right)+\overset{g}{\underset{i=1}{\sum }}f_{i} \end{array}$$

where *F* is the DOFs of the robot, *n* indicates the number of links included in the frame, *g* represents the number of joints, and *f*_*i*_ is the DOFs permitted by joint *i*.

Since *n* = 6, *g* = 7, Σ *f*_*i*_= 15 (the robot contains three spherical joints, two universal joints and two prismatic joints), the DOFs of the parallel mechanism can be obtained by equation 1 as *F* = 3.

### Kinematic Analysis

**Figure 3:**
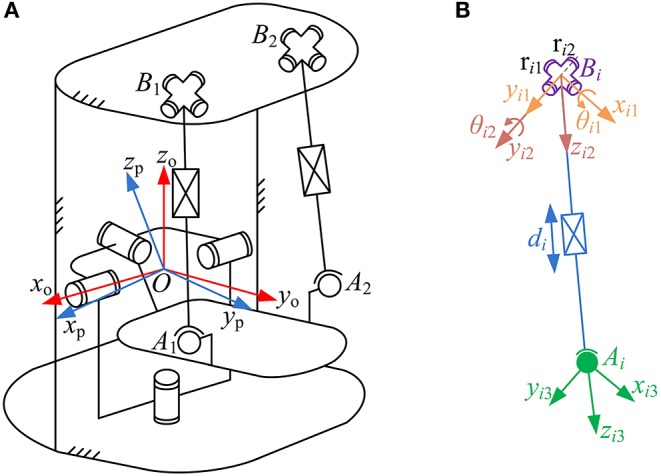
Parametric description of **(A)** the robot and **(B)** kinematic branch.

The schematic diagram of the parallel robot is presented in Figure 3. Several reference frames, i.e., *O-x*_o_*y*_o_*z*_o_, *O-x*_p_*y*_p_*z*_p_, *B*_*i*_*-x*_*i*__1_*y*_*i*__1_*z*_*i*__1_, *B*_*i*_*-x*_*i*__2_*y*_*i*__2_*z*_*i*__2_, and *A*_*i*_*-x*_*i*__3_*y*_*i*__3_*z*_*i*__3_, are established. Reference frames *O-x*_o_*y*_o_*z*_o_ and *O-x*_p_*y*_p_*z*_p_ (Figure 3A), which attached to rotation center *O* of the moving platform, are the base frame and the moving platform frame, respectively. These two reference frames are parallel in their initial configurations. As shown in Figure 3B, a local fixed reference frame *B*_*i*_*-x*_*i*__1_*y*_*i*__1_*z*_*i*__1_ (*i* = 1, 2) and a movable reference frame *B*_*i*_*-x*_*i*__2_*y*_*i*__2_*z*_*i*__2_ (*i* = 1, 2) are both assigned at the center *B*_*i*_ of joint U_*i*_. Axes *x*_*i*__1_, *y*_*i*__2_, *z*_*i*__2_ are collinear to the two revolute axes (r_i1_ and r_i2_) of joint U_*i*_ and the lower link of the kinematic branch, respectively. In the set-up configuration, the axis *y*_*i*__1_ coincides with the axis r_i2_ of joint U_*i*_, and initial angles appear between the frames *B*_*i*_*-x*_*i*__1_*y*_*i*__1_*z*_*i*__1_ and *B*_*i*_*-x*_*i*__2_*y*_*i*__2_*z*_*i*__2_. Moreover, reference frame *A*_*i*_*-x*_*i*__3_*y*_*i*__3_*z*_*i*__3_ (*i* = 1, 2) in joint S_*i*_ location is assigned at the center *A*_*i*_, and the directions of its three coordinate axes coincide with those of the frame *B*_*i*_*-x*_*i*__2_*y*_*i*__2_*z*_*i*__2_. In this paper, due to the three rotational DOFs of the wearable parallel robot, the posture of the moving platform can be described by its orientation with respect to the base.

Considering the characteristics of the constraint branch, the *Z-X-Y* typed Euler angles can be utilized to express the orientation of the moving platform. The angles rotating about axis *z*_p_ (AD/AB), axis *x*_p_ (PL/DO) and axis *y*_p_ (IN/EV) are denoted as γ, α, and β, respectively. The transformation from reference frame *O-x*_p_*y*_p_*z*_p_ to *O-x*_o_*y*_o_*z*_o_, denoted as matrix ***R***_op_, is given as:

(2) $$\begin{array}{l} {\text{R}}_{\text{op}}=\text{R}\left(\gamma \right)\text{R}\left(\alpha \right)\text{R}\left(\beta \right) \end{array}$$

#### Inverse Position Solution

The inverse kinematics problem posed by a parallel mechanism is easy to manage (Li et al., 2018), in which a desired posture of the moving platform is given, and the drive variables can be calculated to achieve this task.

The coordinates of point *A*_*i*_ (*i* = 1, 2) can be computed simultaneously as follows:

(3) Ao i=Rop pAi

(4) $${}^{\text{o}}A_{i}=d_{i}z_{i2}{+}^{\text{o}}B_{i}=d_{i}R_{\text{o}2}{\left(0,\;0,\;1\right)}^{\text{T}}{+}^{\text{o}}B_{i}$$

where °***A***_*i*_ and ^p^***A***_*i*_ are the position vector of point *A*_*i*_ with respect to reference frames *O-x*_o_*y*_o_*z*_o_ and *O-x*_p_*y*_p_*z*_p_, respectively. °***B***_*i*_ (*i* = 1, 2) denotes the position vector of point *B*_*i*_ expressed in reference frame *O-x*_o_*y*_o_*z*_o_. *d*_*i*_ is the displacement of joint P_*i*_, whereas ***z***_*i*__2_ is the direction vector of axis *z*_*i*__2_. The transformation matrix ***R***_o2_ that transfers the coordinates from reference frame *O-x*_o_*y*_o_*z*_o_ to *B*_*i*_*-x*_*i*__2_*y*_*i*__2_*z*_*i*__2_ can be expressed as follows:

(5) $$\begin{array}{l} {\text{R}}_{\text{o}2}={\text{R}}_{\text{o}1}{\text{R}}_{12} \end{array}$$

where ***R***_o1_ and ***R***_12_ represent the transformation matrices between reference frames *O-x*_o_*y*_o_*z*_o_ and *B*_*i*_*-x*_*i*__1_*y*_*i*__1_*z*_*i*__1_, *B*_*i*_*-x*_*i*__1_*y*_*i*__1_*z*_*i*__1_, and *B*_*i*_*-x*_*i*__2_*y*_*i*__2_*z*_*i*__2_, respectively, and are given as follows:

(6) $$\begin{array}{l} {\text{R}}_{\text{o}1}=\left[\begin{array}{ccc} 0 & -1 & 0\\ 1 & 0 & 0\\ 0 & 0 & 1 \end{array}\right],\\ {\text{R}}_{12}=\left[\begin{array}{ccc} \cos\left(\theta_{i\text{2}}\right) & 0 & \sin\left(\theta_{i2}\right)\\ \sin\left(\theta_{i1}\right)\sin\left(\theta_{i2}\right) & \cos\left(\theta_{i1}\right) & -\cos\left(\theta_{i2}\right)\sin\left(\theta_{i1}\right)\\ -\cos\left(\theta_{i1}\right)\sin\left(\theta_{i2}\right) & \sin\left(\theta_{i1}\right) & \cos\left(\theta_{i1}\right)\cos\left(\theta_{i2}\right) \end{array}\right] \end{array}$$

where θ_*i*__1_ and θ_*i*__2_ denote the rotation angles of joint U_*i*_ around axes r_i1_ and r_i2_, respectively.

By substituting the first half of Equation (4) into Equation (3), individual limb length *d*_*i*_ can be mathematically expressed as follows:

(7) $$\begin{array}{l} d_{i}=|{{\text{R}}_{\text{op}}}^{\text{p}}{\text{A}}_{i}{-}^{\text{o}}{\text{B}}_{i}| \end{array}$$

The rotation angle θ_3_ around axis R_1_ can be simply obtained as:

(8) $$\begin{array}{l} \theta_{3}=\gamma \end{array}$$

Equations (7) and (8) give the inverse position solution of the robot.

Substituting the second half of Equation (4) into Equation (3), and using Equations (5–7), the following equation can be derived:

(9) $$\begin{array}{l} \frac{{\text{R}}_{\text{o}1}^{-1}\left({{\text{R}}_{\text{op}}}^{\text{p}}{\text{A}}_{i}{-}^{\text{o}}{\text{B}}_{i}\right)}{\left|{{\text{R}}_{\text{op}}}^{\text{p}}{\text{A}}_{i}{-}^{\text{o}}{\text{B}}_{i}\right|}=\left[\begin{array}{c} \sin\left(\theta_{i2}\right)\\ -\cos\left(\theta_{i2}\right)\sin\left(\theta_{i1}\right)\\ \cos\left(\theta_{i1}\right)\cos\left(\theta_{i2}\right) \end{array}\right] \end{array}$$

Let *k*_*i*1_, *k*_*i*2_, *k*_*i*3_ indicate the three components of the left vector in equation 9, the following equations can be obtained as follows:

(10) $$\begin{array}{l} \theta_{i2}=\arcsin\left(k_{i1}\right),\theta_{i1}=\frac{1}{2}\arccos\left(\frac{k_{i2}^{2}-k_{i3}^{2}}{1-k_{i1}^{2}}\right) \end{array}$$

#### Velocity Jacobian Matrix

According to the schematic diagram of the robot presented in Figure 3, the velocity vector **V**_*A*_*i*__ of center *A*_*i*_ can be written as:

(11) $$\begin{array}{l} {\text{V}}_{A_{i}}=\omega \times \left({{\text{R}}_{0\text{p}}}^{\text{p}}{\text{A}}_{i}\right) \end{array}$$

where **ω** indicates the angular velocity of the moving platform.

Projecting the velocity vector **V**_*A*_*i*__ onto the reference frame *A*_*i*_*-x*_*i*__3_*y*_*i*__3_*z*_*i*__3_ leads to:

(12) $$\begin{array}{l} \left[\begin{array}{c} {\text{V}}_{x_{i3}}\\ {\text{V}}_{y_{i3}}\\ {\text{V}}_{z_{i3}} \end{array}\right]=\left[\begin{array}{c} {\text{x}}_{i3}^{\text{T}}\\ {\text{y}}_{i3}^{\text{T}}\\ {\text{z}}_{i3}^{\text{T}} \end{array}\right]{\text{V}}_{A_{i}} \end{array}$$

where ${\text{x}}_{i3}^{\text{T}}$, ${\text{y}}_{i3}^{\text{T}}$, and ${\text{z}}_{i3}^{\text{T}}$ denote the direction vectors of axes *x*_*i*__3_, *y*_*i*__3_, and *z*_*i*__3_, respectively, and can be written as follows:

(13) $$\begin{array}{l} {\text{x}}_{i3}={\text{x}}_{i2}={\text{R}}_{\text{o}2}{\left(1,\;0,\;0\right)}^{\text{T}},{\text{y}}_{i3}={\text{y}}_{i2}={\text{R}}_{\text{o}2}{\left(0,\;1,\;0\right)}^{\text{T}},\\ {\text{z}}_{i3}={\text{z}}_{i2}={\text{R}}_{\text{o}2}{\left(0,\;0,\;1\right)}^{\text{T}} \end{array}$$

where ***x***_*i*__2_, ***y***_*i*__2_, and ***z***_*i*__2_ represent the respective direction vectors of axes *x*_*i*__2_, *y*_*i*__2_, and *z*_*i*__2_.

The velocity of the linear actuator can be calculated based on the structural feature of the kinematic branch as:

(14) $$\begin{array}{l} {\dot{d}}_{i}=V_{z_{i3}}=z_{i3}^{\text{T}}V_{A_{i}}=z_{i3}^{\text{T}}Q\omega \end{array}$$

where *V*_*z*_*i*3__ indicates the velocity component of **V**_*A*_*i*__ in the axis *z*_*i*__3_ direction, ḋ_*i*_ is the linear velocity of joint P_*i*_. Moreover, coefficient matrix ***Q***of **V**_*A*_*i*__ can be derived as follow:

(15) $$\begin{array}{l} \text{Q}=\left[\begin{array}{ccc} 0 & {\left(\begin{array}{c} 0\\ 0\\ 1 \end{array}\right)}^{\text{T}}\left({{\text{R}}_{\text{op}}}^{\text{p}}{\text{A}}_{i}\right) & -{\left(\begin{array}{c} 0\\ 1\\ 0 \end{array}\right)}^{\text{T}}\left({{\text{R}}_{\text{op}}}^{\text{p}}{\text{A}}_{i}\right)\\ -{\left(\begin{array}{c} 0\\ 0\\ 1 \end{array}\right)}^{\text{T}}\left({{\text{R}}_{\text{op}}}^{\text{p}}{\text{A}}_{i}\right) & 0 & {\left(\begin{array}{c} 1\\ 0\\ 0 \end{array}\right)}^{\text{T}}\left({{\text{R}}_{\text{op}}}^{\text{p}}{\text{A}}_{i}\right)\\ {\left(\begin{array}{c} 0\\ 1\\ 0 \end{array}\right)}^{\text{T}}\left({{\text{R}}_{\text{op}}}^{\text{p}}{\text{A}}_{i}\right) & -{\left(\begin{array}{c} 1\\ 0\\ 0 \end{array}\right)}^{\text{T}}\left({{\text{R}}_{\text{op}}}^{\text{p}}{\text{A}}_{i}\right) & 0 \end{array}\right] \end{array}$$

The angular velocity ${\dot{\theta }}_{3}$ can be simply obtained as follows:

(16) $$\begin{array}{l} {\dot{\theta }}_{3}=\dot{\gamma } \end{array}$$

Combining Equation (14) and Equation (16), the velocity mapping relationship between active joint space and task space can be expressed as follows:

(17) $$\begin{array}{l} \left[\begin{array}{c} {\dot{d}}_{1}\\ {\dot{d}}_{2}\\ {\dot{\theta }}_{3} \end{array}\right]=\left[\begin{array}{c} \begin{array}{c} {\text{z}}_{13}^{\text{T}}\text{Q}\\ {\text{z}}_{23}^{\text{T}}\text{Q} \end{array}\\ \begin{array}{ccc} 0 & 0 & 1 \end{array} \end{array}\right]\omega ={\text{J}}_{\text{o}}^{-1}\omega \end{array}$$

where ***J***_o_ is the original velocity Jacobian matrix of the robot.

Notably, the input end contains two linear motions and one rotational motion, while the output end consists exclusively of rotational motions (i.e., the velocity Jacobian matrix is dimensionally inhomogeneous). Thus, a non-dimensional form (Angeles, 1992) of the homogeneous Jacobian matrix ***J***_v_ is required to be introduced:

(18) $$\begin{array}{l} {\text{J}}_{\text{v}}^{\text{-}1}=\left[\begin{array}{c} \begin{array}{c} {\text{z}}_{13}^{\text{T}}{\text{Q}}^{*}\\ {\text{z}}_{23}^{\text{T}}{\text{Q}}^{*} \end{array}\\ \begin{array}{ccc} 0 & 0 & 1 \end{array} \end{array}\right] \end{array}$$

where:

(19) $$\begin{array}{l} \begin{array}{l} \;Q^{*}=\left[\begin{array}{ccc} 0 & {\left(\begin{array}{c} 0\\ 0\\ 1 \end{array}\right)}^{\text{T}}(R_{\text{op}}{\;}^{\text{p}}A_{i}^{*}) & -{\left(\begin{array}{c} 0\\ 1\\ 0 \end{array}\right)}^{\text{T}}(R_{\text{op}}{\;}^{\text{p}}A_{i}^{*})\\ -{\left(\begin{array}{c} 0\\ 0\\ 1 \end{array}\right)}^{\text{T}}(R_{\text{op}}{\;}^{\text{p}}A_{i}^{*}) & 0 & {\left(\begin{array}{c} 1\\ 0\\ 0 \end{array}\right)}^{\text{T}}(R_{\text{op}}{\;}^{\text{p}}A_{i}^{*})\\ {\left(\begin{array}{c} 0\\ 1\\ 0 \end{array}\right)}^{\text{T}}(R_{\text{op}}{\;}^{\text{p}}A_{i}^{*}) & -{\left(\begin{array}{c} 1\\ 0\\ 0 \end{array}\right)}^{\text{T}}(R_{\text{op}}{\;}^{\text{p}}A_{i}^{*}) & 0 \end{array}\right],\\ {\;}^{\text{p}}A_{i}^{*}=k^{*}\frac{{\;}^{\text{p}}A_{i}}{{\text{r}}_{\text{sp}}}0 \end{array} \end{array}$$

where ${\text{p}}_{i}^{\text{A}}*$ is the position vector of point *A*_*i*_ with respect to the reference frame *O-x*_p_*y*_p_*z*_p_, expressed in non-dimensional form. A scalar r_sp_ indicates the distribution radius of the spherical joint with respect to the moving platform and is utilized to homogenize the original velocity Jacobian matrix (Zanganeh and Angeles, 1997). k^*^ represents the scaling factor between the linear motion and rotational motion (generally, k^*^= 1).

### Performance Indices

#### Reachable Workspace Index

The reachable workspace of an ankle rehabilitation robot must contain the ROM of the HAC summarized (Siegler et al., 1988) in Table 2. Meanwhile, to ensure that the safety issue will not occur in the process of treatment, the maximum allowable workspace (MAW) of the ankle rehabilitation robot should be constrained in a certain range (Table 2). By using the numerical searching method based on derived inverse position solution while considering the stroke constraint of the linear actuator, the feasible points in the reachable workspace of the ankle rehabilitation robot with certain dimension parameters can be obtained, and then the set of the reachable points forms the overall workspace. To evaluate the workspace, a reachable workspace index *I*_RW_ can be written as follows:

(20) $$\begin{array}{l} I_{\text{RW}}=\frac{v_{\text{RW}}}{v_{\text{MAW}}}, \end{array}$$

(21) $$\begin{array}{l} v_{\text{RW}}=\int_{w}dw, \end{array}$$

(22) $$\begin{array}{l} v_{\text{MAW}}=\Delta \alpha \Delta \beta \Delta \gamma \;, \end{array}$$

where *v*_RW_and *v*_MAW_ are the volume of the reachable workspace and the MAW, respectively, Δα, Δβ, and Δγ denote the ranges between the minimum and maximum α, β, and γ, respectively, which can be given as follows:

(23) $$\begin{array}{l} \Delta \alpha =\alpha_{\max}-\alpha_{\min}, \end{array}$$

(24) $$\begin{array}{l} \Delta \beta =\beta_{\max}-\beta_{\min}, \end{array}$$

(25) $$\begin{array}{l} \Delta \gamma =\gamma_{\max}-\gamma_{\min}, \end{array}$$

where α_max_, α_min_, β_max_, β_min_, γ_max_, and γ_min_ can be obtained according to Table 2. The *I*_RW_ can reach values from 0 to 1. The value is equal to 1 (or 0) mean that the robot possesses the largest (or smallest) workspace.

**Table 2:** ROM of the HAC and MAW of the robot.

**Type of motion**	**ROM/(^**°**^)**	**MAW of the robot /(^**°**^)**
Plantarflexion	37.6–45.8	45.0
Dorsiflexion	20.3–29.8	30.0
Inversion	14.5–22.0	22.0
Eversion	10.0–17.0	22.0
Adduction	22.0–36.0	36.0
Abduction	15.4–25.9	36.0

#### Motion Isotropy Index

The inverse value of the condition number of robot's velocity Jacobian matrix, ranges between 0 and 1 (denote singular and isotropic configuration, respectively), is an important local performance index to evaluate the motion isotropy in one posture or over its full workspace of a parallel robot (Wu et al., 2013; Enferadi and Nikrooz, 2017). Its physical meaning can be expressed as a velocity ellipsoid and define as Equation (26). For a rehabilitation device, as many areas as possible in the reachable workspace are desired to possess relatively uniform motion isotropy. That is, condition number's inverse value of most of the feasible points should be closer to 1. To measure the global behavior of the condition number of the robot, a motion isotropy index *I*_MI_ can be presented via computing the average of the inverse value of the condition number within the reachable workspace, and is written as follows:

(26) $$\begin{array}{l} {\dot{\text{S}}}^{\text{T}}\dot{\text{S}}=\omega^{\text{T}}{\left({\text{J}}_{\text{v}}{\text{J}}_{\text{v}}^{\text{T}}\right)}^{-1}\omega \leq 1\;, \end{array}$$

(27) $$\begin{array}{l} I_{\text{MI}}=\frac{\int_{w}\eta_{\text{J}}dw}{v_{\text{RW}}}, \end{array}$$

(28) $$\begin{array}{l} \eta_{\text{J}}=l_{\text{vsp}}/l_{\text{vlp}} \end{array}$$

where $\dot{\text{S}}\text{=}{\left({\dot{d}}_{1},\;{\dot{d}}_{2},\;{\dot{\theta }}_{3}\right)}^{T}$; *w* denotes the reachable workspace of the ankle rehabilitation robot; η_J_ is a local index indicating the inverse value of the condition number of robot's velocity Jacobian matrix in a given posture within the reachable workspace; *l*_vlp_ and *l*_vsp_ are the lengths of the long and short principal axes of the velocity ellipsoid, respectively. The value range of the *I*_MI_ is between 0 and 1, and the value of which is desired to be larger.

#### Force Tansfer Index

As a human-robot system for ankle rehabilitation, the force is required to be transferred from robot's active joint space to patient's ankle space as sufficient torque which is an important condition for an ankle rehabilitation robot to achieve passive/active treatment. A force unit sphere ***f***^T^***f*** ≤ 1 is set up in active joint space. Subsequently, this sphere can be transferred into task space as a force ellipsoid via the force mapping relationship, and can be defined as follows:

(29) $$\begin{array}{l} f=J_{\text{f}}\tau =J_{\text{v}}^{\text{T}}\tau , \end{array}$$

(30) $$\begin{array}{l} f^{\text{T}}f\text{=}\tau^{\text{T}}\left(J_{\text{f}}^{\text{T}}J_{\text{f}}\right)\tau =\tau^{\text{T}}\left(J_{\text{v}}J_{\text{v}}^{\text{T}}\right)\tau \leq 1, \end{array}$$

where $\text{f}\text{=}{\left(f_{1},f_{2},\tau_{3}\right)}^{\text{T}}$ and $\tau \text{=}{\left(\tau_{\;\alpha \;},\tau_{\;\beta \;},\tau_{\;\gamma \;}\right)}^{\text{T}}$; *f*_1_ and *f*_2_ are the driving forces of joints P_1_ and P_2_, and τ_3_ is the driving torque of joint R_1_; τ_α_, τ_β_, and τ_γ_ indicate the torques applied on the axes of PL/DO, IN/EV, and AB/AD, respectively; ***J***_f_ denotes the force Jacobian matrix, and is the transpose of Jacobian matrix ***J***_v_.

The robot possesses a better (or worse) force transfer performance along a particular operation direction when the length of the force ellipsoid's radius along the directional vector is longer (or shorter). Moreover, the long (or short) principal directions of the force ellipsoid means the greatest (or least) force transfer performance. Thus, the length *l*_fsp_ of the short principal axis of the force ellipsoid can be regarded as a local evaluation index of force transfer performance, and the corresponding global force transfer index *I*_FT_ is given as follows:

(31) $$\begin{array}{l} I_{\text{FT}}=\frac{\int_{w}l_{\text{fsp}}dw}{v_{\text{RW}}.} \end{array}$$

#### Maximum Torque Index

To evaluate the force capability of the ankle rehabilitation robot while considering the real physical capability of robot's actuators, a set ***T***_τ_ (or convex polyhedrons) of allowable forces and torques of the actuators should be defined based on the force Jacobian matrix in the task space. The radius *r*_is_ of the inscribed sphere contained in the set indicates the largest real torque that can be realized by the ankle rehabilitation robot along all directions in the ankle space, i.e., this radius reflects the maximum torque in a given posture. According to the aforementioned analysis and the force mapping relationship, the set ***T***_τ_, and a global maximum torque index *I*_MT_ of the ankle rehabilitation robot can be written as follows:

(32) $$\begin{array}{l} T_{\tau }=\left\{\begin{array}{ccc} \tau | & \tau ={\left(J_{\text{f}}\right)}^{-1}f & f\in T_{f} \end{array}\right\}\;, \end{array}$$

(33) $$\begin{array}{l} T_{f}=\left\{f|\;|f_{i}|\leq f_{i\max}\;i=1,2;\;|\tau_{\text{3}}|\leq \tau_{3\max}\right\}\;, \end{array}$$

(34) $$\begin{array}{l} I_{\text{MT}}=\frac{\int_{w}l_{\text{ris}}dw}{v_{\text{RW}}}\;, \end{array}$$

where ***T***_τ_ is the generalized set of torques in the ankle space; ***T***_*f*_ is the allowable forces and torques of the actuator; *l*_ris_ is a local index denoting the length of the *r*_is_, and is actually a local performance index. Without loss of generality, the force and torque limits of the driven system are assumed to be *f*_1max_ = *f*_2max_ = τ_3max_ = 1. Thus, the *I*_MT_ ranges from 0 to 1, a larger (or smaller) value of *I*_MT_ indicates a better (or worse) force capability of the ankle rehabilitation robot.

## Results

### Mechanical Design

**Figure 4:**
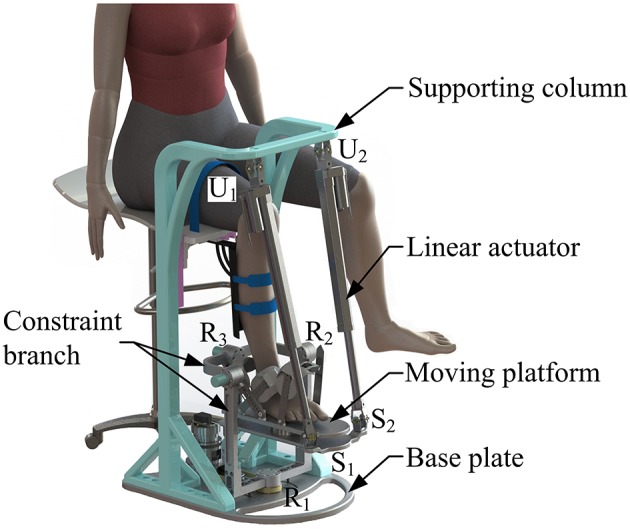
Wearable parallel robot.

Based on the proposed kinematic configuration (i.e., 2-UPS/RRR parallel mechanism) and wearable design concept, the mechanical design of the wearable parallel robot (Figure 4) is detailed. Two sub-systems, i.e., a basic machine-drive system and a multi-model position/force data collection system, constitute the whole physical system of the robot.

**Figure 5:**
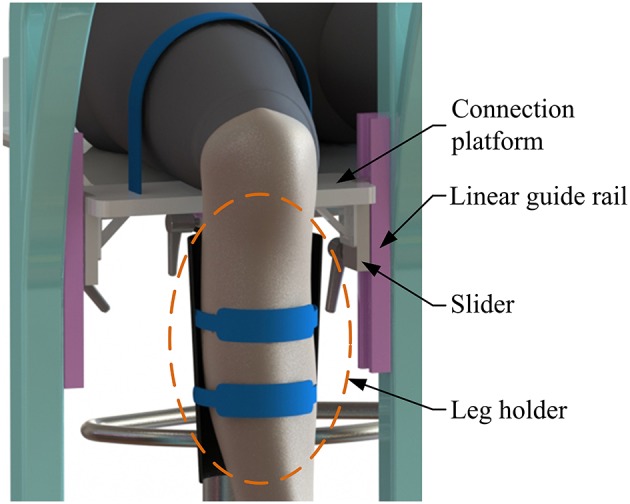
Lower-limb binding mechanism.

**Figure 6:**
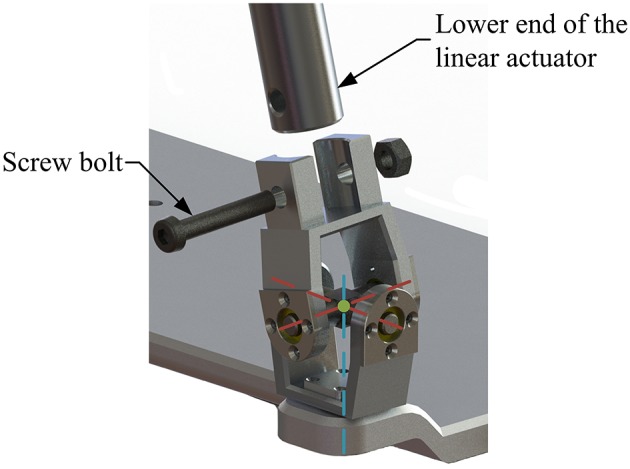
Joint S_1_ (S_2_).

**Figure 7:**
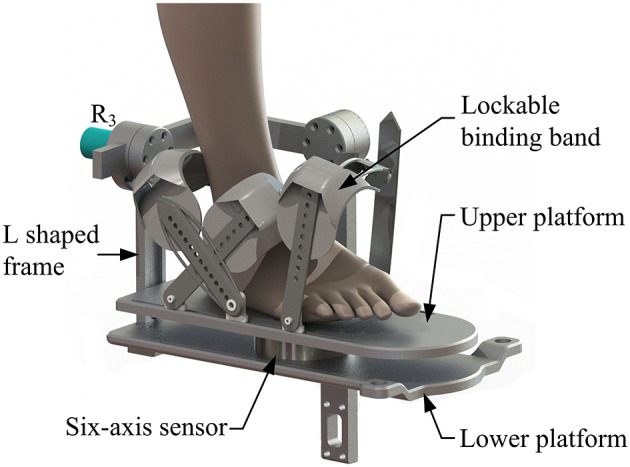
Moving platform.

**Figure 8:**
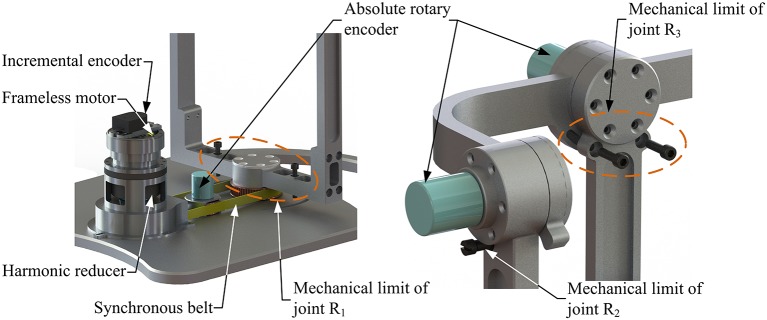
Mechanical limits, joint R_1_ and absolute rotary encoders.

The machine-drive system exhibits a parallel main structure, in which two kinematic branches and one constraint branch both connect the base to the moving platform. The base consists of a base plate and a supporting column. An adjustable lower-limb binding mechanism is established between the supporting column to maintain the stability of the lower-limb during rehabilitation and accommodate patients with different body sizes (Figure 5), double linear guide rails with double-slider, single-connection platform and locking function are employed to fix the thigh and adjust the up/down position of the patient, while the calf is fixed by a special leg holder and its forward/backward position can be fine-tuned by single linear guide rail with single-slider and locking function. Subsequently, the base and two identical kinematic branches are connected via the joint U_1_ and U_2_. Two linear actuators (CAHB-10, SKF, Sweden) are employed as joint P_1_ and P_2_ to adjust the lengths of the kinematic branches from 413 to 713 mm. As illustrated in Figure 6, joint S_1_ (S_2_) that located below the joint U_1_ (U_2_), and P_1_ (P_2_) is equivalent to a universal joint and a revolute joint with three axes intersecting at the same point, this combination design can reduce the cost and realize free-interference on workspace. Moreover, by loosening the screw bolt connecting the lower end of the linear actuator and joint S_1_ (S_2_), the kinematic branches can be separated from the constraint branch (i.e., the 2-UPS/RRR parallel robot is translated into an RRR serial robot). As shown in Figure 7, three lockable binding bands secure the patient's foot to the upper part of the moving platform (i.e., the upper platform) without large misalignment during combined motion. In addition to joint S_1_ and S_2_, the lower platform is also connected to joint R_3_ via an “L” shaped frame. As the active vertical-revolute joint of the constraint branch, joint R_1_ is driven by a servo motor (Figure 8), i.e., the combination of a frameless motor (KBM, Kollmorgen, America) and an incremental encoder (HKT30-301, REP, China), and is transmitted via a harmonic reducer (CSG-17-100, HarmonicDrive, Japan). Notably, the distance between the moving platform and the base plate determines the height of the patient's seat, and a higher seat may produce fear emotions that lead to negative treatment results on patient. Thus, to reduce the height of the moving platform, the axis R_1_ and its driving unit (i.e., the servo motor and the incremental encoder) are arranged in parallel and are connected to each other by a synchronous belt with a 1:1 reduction ratio. Additionally, as shown in Figure 8, screw bolts are installed as mechanical limits on joint R_1_, and two suspended revolute joints (i.e., joints R_2_ and R_3_) for safety. As mentioned above, the limits for the rotation angles are set according to the MAW in Table 2, and the maximum allowable angles of the PL/DO (α_max_/α_min_), IN/EV (β_max_/β_min_), and AD/AB (γ_max_/γ_min_) are set at 45°/30°, 22°/22°, and 36°/36° to ensure that the robot is suitable for both the left and right foot.

**Figure 9:**
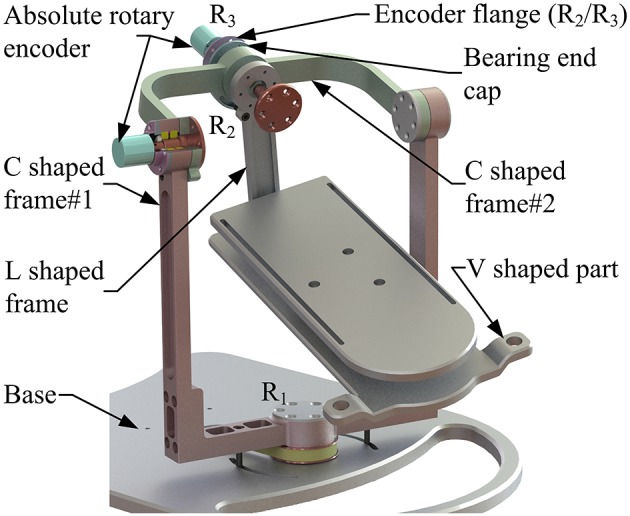
Detailed view of joints R_2_ and R_3_.

A multi-model position/force data collection system is installed in the robot to realize various rehabilitation strategies, including ROM treatment based on position control, strength treatment based on impedance control and proprioceptive treatment base on intention recognition. Specifically, as illustrated in Figure 8, three absolute rotary encoders (HAN28U5, China) are arranged to measure the rotation angles of joints R_1_, R_2_, and R_3_ (i.e., real-time position information of the moving platform); two of them are connected in series with joints R_2_ and R_3_, as shown in the detailed view (Figure 9), joints R_2_ and R_3_ possess similar structure: the base, “C” shaped frame#1, “C” shaped frame#2, and “L” shaped frame are arranged in sequence, the latter rotates with respect to the former, and the “L” shaped frame drives the moving platform together with the “V” shaped part of the lower platform; another one is placed on the base plate and arranged in parallel with joint R_1_. Additionally, the force/torque information of the rehabilitation process, i.e., the interaction force and torque between the foot and moving platform, can be collected by a six-axis sensor installed between the upper and lower platform (Figure 7). In general, the encoders and sensor form a complete information collection system, which can produce real-time feedback in the process of treatment and lay a foundation for various control schemes and rehabilitation strategies.

### Performance Evaluation

The geometrical parameters of the wearable parallel robot are presented in Table 3, where the absolute values of the coordinates of joint U_*i*_ are expressed with respect to reference frame *O-x*_o_*y*_o_*z*_o_, the absolute values of the coordinates of joint S_*i*_ are expressed with respect to reference frame *O-x*_p_*y*_p_*z*_p_.

**Table 3:** The geometrical dimensions of the robot.

**Robotic dimensions**	**Absolute values** **of coordinates**		
	**X**	**Y**	**Z**
Center *B_*i*_* of joint U*_*i*_*	120 mm	70 mm	520 mm
Center *A_*i*_* of joint S*_*i*_*	90 mm	215 mm	107 mm

**Figure 10:**
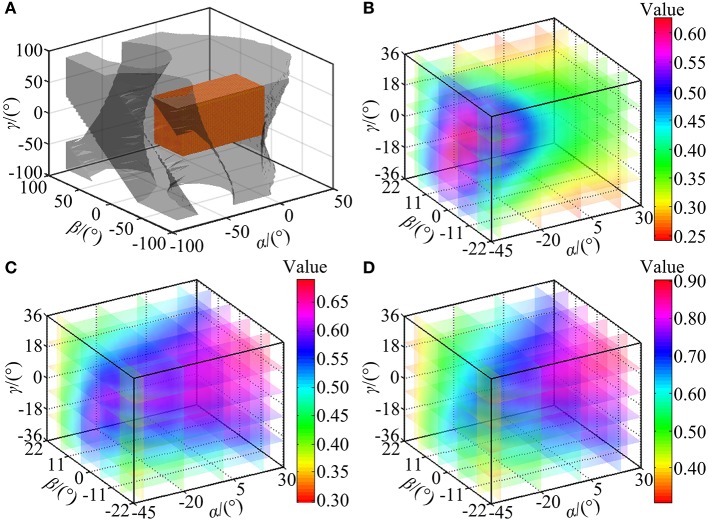
Performance indices calculation. **(A)** The reachable workspace of the robot. **(B)** Trend in value of η_J_. **(C)** Trend in value of *l*_fsp_. **(D)** Trend in value of *l*_ris_.

The reachable workspace (the set of the solid points) of the wearable parallel robot is calculated in Figure 10A. For this robot, the constraint condition is the stroke constraint of the linear actuators and the arrangement of the mechanical limits. According to the calculation results, the appearance of the reachable workspace is a cube, suggesting that the robot can reach any posture in MAW, i.e., the volume of the reachable workspace is equal to that of the MAW, and thus *I*_RW_ = 1. Additionally, when the mechanical limits are removed, the workspace of the robot is represented by a high transparency shadow (Figure 10A) which covers the set of the solid points, indicating that the mechanical limits can effectively restrict the workspace to a safe range.

**Figure 11:**
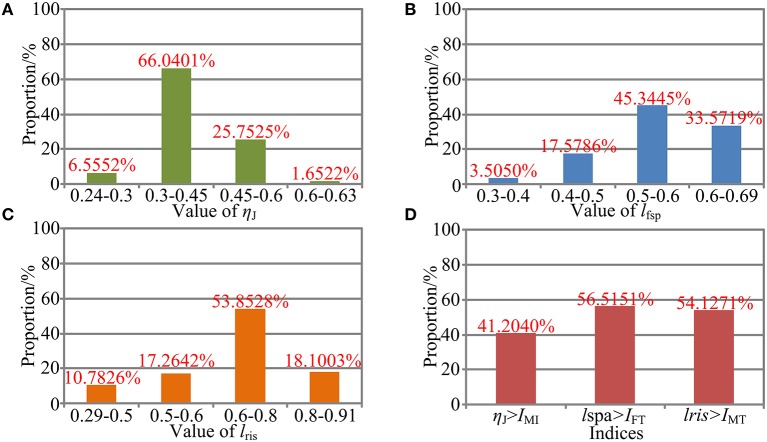
Performance evaluation. **(A)** Performance distribution of η_J_. **(B)** Performance distribution of *l*_fsp_. **(C)** Performance distribution of *l*_ris_. **(D)** Performance comparison between the local indices and the global indices.

The η_J_, *l*_spa_, and *l*_ris_ are evaluated within the calculated reachable workspace. Subsequently, trends of their corresponding values are illustrated in Figures 10B–D, respectively, with values represented by the color map shown in the color bar and trends represented by the color variation in the color map. The distribution volume proportions of the corresponding values in the reachable workspace are shown in Figures 11A–C, while the proportion that indices η_J_, *l*_fsp_, and *l*_ris_ superior to indices *I*_MI_, *I*_FT_, and *I*_MT_ is shown in Figure 11D.

As shown in Figure 10B, the values of η_J_ change smoothly with no mutation within overall reachable workspace, indicating that the robot has no singularity configuration. Moreover, the robot exhibits better motion isotropy performance in the central part of the reachable workspace, since the values of the η_J_ are relatively small in the boundary area and increases gradually toward the central section. As shown in Figure 11A, the value of η_J_ is mainly distributed between 0.3 and 0.6, and the minimum value, varies from 0.24 to 0.3, accounted for 6.5552% in the reachable workspace. Thus, the robot is sufficiently kinematically isotropic for ankle rehabilitation.

Analogous to η_J_, both *l*_fsp_ and *l*_ris_ possess better performance in the central part (Figures 10C,D). As illustrated in Figures 11B,C, the values of *l*_fsp_ and *l*_ris_ are mainly distributed between 0.5 and 0.69, 0.6, and 0.91, respectively. Moreover, the proportions of the worst-performing postures of *l*_fsp_ and *l*_ris_ are 3.5050 and 10.7826%. Thus, the robot possesses high force transfer performance and large maximum torque performance, especially in the central part.

The values of *I*_MI_, *I*_FT_, and *I*_MT_ are calculated as *I*_MI_ = 0.5573, *I*_FT_ = 0.5565, and *I*_MT_ = 0.6744, demonstrating sufficient global performances. As shown in Figure 11D, the proportion of η_J_, *l*_fsp_, and *l*_ris_ exceeded *I*_MI_, *I*_FT_, and *I*_MT_ in magnitude are 41.2040, 56.5151, and 54.1271%, mainly located in the central section (i.e., main treatment area), suggesting that most postures within the reachable workspace are well-performed enough (although the proportion of η_J_ superior to indices *I*_MI_ is failure to reach 50%, most postures that do not meet the condition exceed 0.5 in magnitude, as shown in Figure 10B).

**Figure 12:**
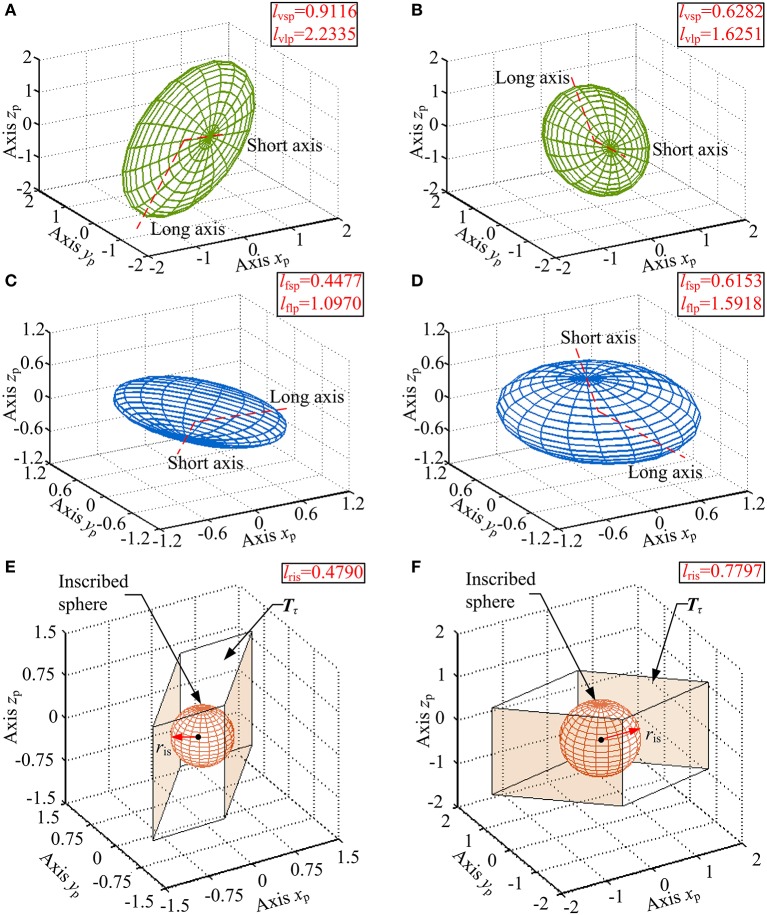
**(A,B)** Velocity ellipsoids, **(C,D)** force ellipsoids, **(E,F)** sets ***T***_τ_ and their inscribed spheres for [α = −30°, β = 20°, γ = 5°], [α = 10°, β = −10°, γ = −2°] configurations.

To verify the calculation correctness and obtain a detailed view, velocity ellipsoids, force ellipsoids, sets ***T***_τ_ and their inscribed spheres in two stochastic configurations ([α = −30°, β = 20°, γ = 5°], [α = 10°, β = −10°, γ = −2°]) of the reachable workspace are provided in Figure 12. Figures 12A–D report that due to the duality between the velocity ellipsoid and the force ellipsoid (Chiu, 1988), the principal axial directions of the two ellipsoids are coincident and the lengths, i.e. *l*_vsp_ and *l*_flp_ (the length of the long principal axis of the force ellipsoid), *l*_vlp_ and *l*_fsp_, are reciprocal. Index η_J_ are calculated as 0.4081 and 0.3866 in these two configurations, while index *l*_spa_ are obtained as 0.4477 and 0.6153, respectively. Figures 12E,F indicate that the cube in active joint space maps to an irregular polyhedron in task space with an inscribed sphere tangent to the colored surfaces, and index *l*_ris_ are, respectively calculated as 0.4790 and 0.7797.

## Discussions

We introduced a novel robot with the features of a wearable design concept and parallel platform-based form. The salient advantages of this robot are its simple configuration and safety guarantee. Moreover, we evaluated and analyzed the performance of this robot within the overall reachable workspace. Results showed that the proposed robot possesses sufficient motion isotropy, high force transfer performance and large maximum torque performance.

In comparison with the ankle rehabilitation robot applying redundant actuation, by adopting a simple kinematic configuration (i.e., 2-UPS/RRR parallel mechanism) as the main mechanical structure, actuator non-redundancy and easy operation can be realized on this wearable parallel robot, and then the cost of the robot manufacturing, the difficultly of the control system development, and the burden of the therapists can also be effectively reduced. Additionally, according to the performance evaluation and analysis, the proposed robot satisfies the conditions of performing ankle rehabilitation treatment for patients.

This wearable parallel robot is designed with several safety precautions to protect the patients from secondary injury in the process of treatment. Specifically, this robot possesses the lowest moving platform under the premise of meeting the ROM requirement, and then correspondingly reduces the height of the patient's seat. An excessively high seat increases the risk of injury and easily results in patients' contravene mood. Moreover, the arrangement of the lower-limb binding mechanism and three lockable binding bands, respectively fix the thigh/calf and the foot with the base and the moving platform. With this wearable design concept, the foot moves in the form of platform-base with respect to the lower-limb, and thus avoiding the coupled foot-shank motion. Additionally, the supporting column is narrow in width to allow the patient to place the non-rehabilitation leg conveniently. Demountable mechanical limits are implemented to constrain the workspace of the robot in a safe range.

In terms of information collection, the proposed robot is equipped with multi-model position/force data collection system containing a six-axis sensor and three absolute rotary encoders. Based on the collected kinematic and dynamic information, passive treatment mode and active treatment mode can be achieved. By separating the spherical joint from the upper end of the linear actuators, the 2UPS/RRR parallel robot can be translated into an RRR serial robot. Therapists can bind the patient's foot on the moving platform in advance and plan the treatment trajectory by manually moving the RRR serial robot according to the joint characteristics and the severity of impairment among different patients. The trajectory can be recorded by the three absolute rotary encoders, and the robot can provide repetitive treatment on the basis of this trajectory.

It should be highlight that the indices are not applied to design the geometrical parameters of the mechanism in this study. A low height of the moving platform, a narrow width of the overall mechanism and a sufficient volume of the reachable workspace are more considered at current stage. Moreover, by applying the transmission angular theory and screw theory to evaluate the motion/force transmissibility (i.e., essential function) of the parallel mechanism, a more convincing kinematic performance evaluation system (Liu et al., 2008; Wu et al., 2010, 2011; Xie et al., 2010) was proposed compared with the utilization of the Jacobian matrix in evaluation of parallel mechanism. Based on the defined motion/force transmission indices, dimensional synthesis (optimal design) was also carried out using performance charts. In future studies, dimension optimal design according to the aforementioned method will be carried out before prototype construction. Additionally, based on the multi-model position/force data collection system, future work could go in the direction of the development of the control schemes to achieve various rehabilitation protocols.

## Conclusion

This paper presented a novel wearable parallel robot for ankle rehabilitation in which the intended simple configuration is determined according to the HAC anatomy and safety consideration. Based on the performance evaluation, the proposed robot possesses relatively uniform motion isotropy, high force transfer performance and large maximum torque performance within a large reachable workspace. Equipped with a multi-model position/force data collection system, both passive and active treatment mode can be achieved. And the robot has the potential to be used for the rehabilitation treatment of ankle disabilities.

## Data Availability Statement

The datasets analyzed in this article are not publicly available. Requests to access the datasets should be directed to dongmj@bjut.edu.cn.

## Author Contributions

SZ and JL conceived and designed this study. SZ, XZ, WF, and YK performed the mechanical design and performance evaluation. SZ wrote the paper. MD reviewed and edited the manuscript. JL also made a contribution to the edition of the manuscript. All authors had read and approved the manuscript.

### Conflict of Interest

The authors declare that the research was conducted in the absence of any commercial or financial relationships that could be construed as a potential conflict of interest.
